# Internet Versus Mailed Questionnaires: A Randomized Comparison

**DOI:** 10.2196/jmir.6.3.e29

**Published:** 2004-09-15

**Authors:** Philip Ritter, Kate Lorig, Diana Laurent, Katy Matthews

**Affiliations:** ^1^Stanford University School of MedicinePalo Alto CAUSA

**Keywords:** Questionnaire design, evaluation, methodology, instruments, Internet

## Abstract

**Background:**

The use of Internet-based questionnaires for collection of data to evaluate patient education and other interventions has increased in recent years. Many self-report instruments have been validated using paper-and-pencil versions, but we cannot assume that the psychometric properties of an Internet-based version will be identical.

**Objectives:**

To look at similarities and differences between the Internet versions and the paper-and-pencil versions of 16 existing self-report instruments useful in evaluation of patient interventions.

**Methods:**

Participants were recruited via the Internet and volunteered to participate (N=397), after which they were randomly assigned to fill out questionnaires online or via mailed paper-and-pencil versions. The self-report instruments measured were overall health, health distress, practice mental stress management, Health Assessment Questionnaire (HAQ) disability, illness intrusiveness, activity limitations, visual numeric for pain, visual numeric for shortness of breath, visual numeric for fatigue, self-efficacy for managing disease, aerobic exercise, stretching and strengthening exercise, visits to MD, hospitalizations, hospital days, and emergency room visits. Means, ranges, and confidence intervals are given for each instrument within each type of questionnaire. The results from the two questionnaires were compared using both parametric and non-parametric tests. Reliability tests were given for multi-item instruments. A separate sample (N=30) filled out identical questionnaires over the Internet within a few days and correlations were used to assess test-retest reliability.

**Results:**

Out of 16 instruments, none showed significant differences when the appropriate tests were used. Construct reliability was similar within each type of questionnaire, and Internet test-retest reliability was high. Internet questionnaires required less follow-up to achieve a slightly (non-significant) higher completion rate compared to mailed questionnaires.

**Conclusions:**

Among a convenience sample recruited via the Internet, results from those randomly assigned to Internet participation were at least as good as, if not better than, among those assigned mailed questionnaires, with less recruitment effort required. The instruments administered via the Internet appear to be reliable, and to be answered similarly to the way they are answered when they are administered via traditional mailed paper questionnaires.

## Introduction

The purpose of the study was to test the reliability for Internet use of 16 existing self-report instruments that can be used in Internet health services research and intervention studies. Participants in the study were randomized to answer questionnaires on the Internet or via a mailed (paper-and-pencil) questionnaire.

Although we and others have been using these instruments in Internet-based studies for a few years [1], neither these nor most similar instruments had previously been tested for Internet use. This lack of psychometric testing might cause some to question the outcomes of Internet-based health studies.

In searching the literature, we found a number of studies testing particular Internet measures, especially within the field of psychology. For example, Lin et al (2003) studied a measure of self-assessment of depression [2], while Farvolden et al (2003) looked at screening for clinical depression [3]. An increasing number of studies have directly compared paper-and-pencil–administered questionnaires with an Internet-mediated questionnaire. In a study of a 13-item quality of life scale, the Foundation for Accountability [4] found that while there was some variation in individual items, the mean scores for mail and Internet collection were similar. Buchanan and Smith (1999) [5] compared a Web-based personality assessment to a paper-and-pencil version, and using confirmatory factor analyses, found similar psychometric properties in the two tests. Davis (1999) [6] also compared Web and paper-and-pencil versions of a personality measure (rumination), and concluded that “findings from Web-based questionnaire research are comparable with results obtained using standard procedures.” Riva et al (2003) [7] compared attitudes regarding the Internet and concluded that if sampling control and validity assessment is provided, the Internet is a suitable alternative to traditional paper-based methods. Joinson (1999) reported that both anonymity and Web usage (compared to paper-and-pencil) resulted in lower scores on a social desirability measure [8]. And Buchanan (2003) [9] reported that even when Internet-based versions of instruments are reliable and valid, normative data from paper-and-pencil versions may not always compare directly with Internet-mediated psychological testing. A recent overview entitled “Using the Internet for Surveys and Health Research” (Eysenbach & Wyatt 2002, [10]), barely touched on instruments (referring readers to the Quality of Life Instruments Database at Quality of Life Instruments database [11]) and did not discuss validity or reliability of Internet-based questionnaires. Although progress is being made, there remains a need to evaluate Internet versions of most of the health-behavior and outcome instruments useful to researchers evaluating patient intervention programs.

Information is presented on the distributions of the responses using both methods of questionnaire delivery, as well as on the differences between the two sets of responses. The intent is to allow researchers to make an informed decision as to whether each variable is appropriate for Internet use when compared to use via traditional mailed paper self-report questionnaires.

## Methods

### Sample

Over a period of two months, subjects were recruited via the Internet using messages on health discussion groups, community servers, Web-site links, medical e-newsletters, and online support groups. Potential subjects were invited to visit a study Web site and thus all subjects had Internet access. Seven hundred and ninety-one potential subjects expressed interest by leaving contact information at the project Web site, and were invited to participate. Of these, 462 agreed to proceed, were randomized, and were either sent a paper questionnaire or invited to return to a Web site to complete the questionnaire online. Ultimately, 397 were enrolled and filled out questionnaires. We compared the refusal rates of those randomized to the Internet versus those randomized to mailed questionnaires using chi-squares. We also examined the amount of follow-up required for each group.

### Instruments

Information was collected on 16 self-report instruments and well as on demographic variables and types of disease conditions. These instruments have been used extensively in our and others' research, and their mailed paper questionnaire version responses have been previously examined and validated (eg, Lorig et al 1996 [12], also see the research instruments page of the Stanford University Patient Education Research Center's Web site [13]). The criteria for choosing instruments were that they 1) had previously been validated, 2) represented key outcome in studies of one or more chronic conditions, 3) had been used in past studies, 4) were relatively short, and 5) were sensitive to change in the range of .3 effect size. The variables selected were the following instruments.

- Self-Rated Health (1 item). This item comes from the National Health Survey and has been found to be predictive of future health status (Idler & Angel, 1990) [14].
- Health Distress measures worry and concern caused by chronic illness (5 items) (Lorig et al, 1996) [12].
- Number of times per week practice mental stress management and relaxation techniques (1 item) (Lorig et al, 1996) [12].
- Health Assessment Instrument measures disability and is used in the National Health Survey (20 items) (Fries et al, 1980) [15].
- Illness Intrusiveness. Instrument measures how chronic illness affects role function in 5 domains: physical well being and diet, work and finances, marital, sexual and family relations, recreational and social relations, other (13 items) (Devins, 1990) [16].
- Activity limitations, measures role function (4 items) (Lorig et al, 1996) [12].
- Visual numeric instruments for pain, shortness of breath, and fatigue are adaptations of visual analogue instruments that have been found to be easy for subjects to complete (4 items) (González et al, 1995) [17].
- Self-efficacy for managing chronic disease measures the confidence one has in managing chronic conditions and has been found to be predictive of future health status (5 items) (Lorig et al, 1996) [12].
- Self-reported exercise measures minutes/week of aerobic (5 items) and minutes/week stretching and strengthening exercise (1 item) (Lorig et al, 1996) [12].
- Health care utilization (MD visits, hospitalization, hospital days, ER visits) (Lorig et al, 1996; Ritter et al, 2001) [12,18].

Many of the instruments tested were developed by the authors, and all are available for free public use. Detailed information and paper questionnaire-based psychometrics for each of the instruments can be found at the Stanford University Patient Education Research Center Web site [13].

### Data Analyses

We first checked to see if the randomization process had been successful by compared the demographic and disease variables using t-tests. The means for the 16 instruments were then compared using t-tests, Wilcoxin, and analyses of covariance (ANCOVAs). ANCOVAs were run controlling for demographic variable and for the disease variables that were found to differ between the two groups. Confidence intervals were also computed to provide a sense of how much overlap there might be between the answers from the two randomized groups. This information is presented in a way that allows an informed researcher to determine if a particular instrument is appropriate for Internet use and for comparison to results obtained from a traditional paper questionnaire. The standard .05 criterion for determining if there is a significant difference may not be appropriate when one is asserting that there is likely little difference. That criterion is intended to avoid the error of claiming there is a difference when it may only be the result of statistical fluctuation (type I error). But we also wish to avoid the error of claiming there is no difference when there may well be (type II error). Thus we also discuss trends (p=.05 to .10) and slight trends (p=.10 to .20) in case these may indicate a real, albeit small, difference in how the instruments are answered using the two methods.

For multi-item instruments, internal consistency reliability was computed separately within the paper questionnaire and within the Web-based questionnaire groups using Cronbach alpha.

A separate sample was used to compute test-retest correlations. A group of subjects enrolled in an online chronic disease self-management workshop was asked to return to the Web site to fill out a second questionnaire one day after completing a 12-month follow-up questionnaire as part of their study participation. Thirty subjects completed the second questionnaire within one week of completing the first questionnaire. The results of the two sets of answers to the 16 instruments were compared using both Pearson and Spearman correlations.

All subjects received a $10.00 Amazon.com certificate for their participation.

## Results

Four hundred and sixty-two people with chronic disease were invited to participate. If they did not return a mailed questionnaire or fill out the Internet questionnaire after approximately 10 days, they were sent a postcard or follow-up email. As might be expected, many of the mailed questionnaires were not returned within 10 days, and 63.6% were sent a follow-up postcard. Only 27.3% of those randomized to the Internet required a follow-up email (chi square=<.0001). After an additional 10 days with no response, a phone call was made to those randomized to the mailed questionnaire and a reminder email was sent to the Internet group. Of those randomized to mailed questionnaires, 29.4% required a follow-up phone call, and of those assigned to Internet participation, 16.0% required a reminder email (chi square=.0006). Finally, after an additional one to two weeks, a follow-up letter went to 20.3% of those randomized to mail, and a second email was sent to 13.4% of those randomized to the Internet (chi square=.064). Of the participants randomized to mail, 83.1% eventually returned their questionnaires, as did 87.5 % of those randomized to the Internet (chi square=.189). This return rate is defined as those who actually returned their questionnaires or who logged on and filled out a questionnaire divided by the number who agreed to participate and were randomized.

When we compared the demographic characteristics of those who answered their questionnaires on the Internet versus those who used mailed paper questionnaires, we found two slight differences (Table 1). The Internet subjects were slightly more likely to be married than the paper questionnaire subjects (P=.043). In addition, the mailed questionnaire subjects had a slightly higher incidence of asthma (p=.096). Thus, the asthma and marital status variables, as well as the other demographic variables, were included as covariates in the ANCOVA models.

Table 2 presents the means for the 16 instruments and the probability that there are differences in those means, comparing those who answered questionnaires on the Internet with those who used mailed paper questionnaires. Only shortness of breath showed a trend toward being statistically significantly different when the two groups were compared using t-test (p=.074) or Wilcoxin test (p=.081). However, there was also a trend toward the mailed questionnaire sample having higher levels of asthma (Table 1), and when ANCOVAs were used to control for asthma and other demographic variables, the significance rose to p=.254.

Although there were no other differences approaching significance, there were slight trends (less than .20) for ER Visits (p=.146) and health distress (p=.116). The ER visits are very skewed in distribution (with most participants reporting 0), and when the differences were tested using Wilcoxin, the p value rose to .330. Health distress continued to show a slight trend toward a difference, regardless of the test (p=.111 with Wilcoxin, p=.193 from ANCOVAs).

Table 2 also provides information on the distributions of each variable. Standard deviations and 5% to 95% confidence intervals for each randomized group are shown. These illustrate the considerable overlap found between those answering the questionnaires using Web questionnaires and those using mailed questionnaires for all instruments with the possible exception of the Shortness of Breath Visual Numeric Scale.

Internal consistency reliability (Cronbach alpha) was nearly identical for multi-item instruments, whether administered via the Internet or by paper questionnaire (Table 3).

Table 3 also includes the test-retest reliability scores. We saw consistently high correlations, whether Pearson or Spearman correlations were used. This is in spite of the fact that some items such as pain, fatigue and shortness of breath were asked regarding the preceding two weeks, and could have been expected to change in the time between the two questionnaires. The relaxation variable specifically asked about the preceding week (How many times did you do mental stress management or relaxation techniques in the last week?), and might have been expected to produce lower test-retest correlations, which it does.

**Table 1:** Demographic and disease variables

Variable	Percent or Mean (Standard Deviation)		Probability of Difference p (t-test)
	Web N=205	Questionnaire N=192	
Percent male	29.8%	25.7%	.364
**Mean years of education** % less than 12 years % 12 years % 13-15 years % 16 years % more than 16 years	16.1 (3.41) range: 3-23 2.9% 8.2 31.7 19.5 37.6	15.7 (3.54) range: 1-23 4.7% 15.1 27.1 20.3 32.8	.119
Mean age	45.9 (14.3) range: 19-89	44.6 (13.5) range: 19-82	.369
% Non Hispanic White % Black % Hispanic % Asian Ethnic category	79.0% 4.9 6.8 5.4	72.3% 5.2 7.9 11.1	.117 (chi-square, p= .324)
% Married	58.1%	47.9%	.043
% with Diabetes	26.3%	26.6%	.960
% with Hypertension	29.8%	33.9%	.382
% with Asthma	20.0%	27.1%	.096
% with COPD or other lung disease	14.2%	14.1%	.981
% with heart disease	9.8%	13.0%	.468

**Table 2:** Comparison of tested variables

Variable	Internet N=205			Mailed Questionnaire N=192			Probability of Difference		
	Mean (Standard Deviation)	Confidence Intervals	Observed Range	Mean (Standard Deviation)	Confidence Intervals	Observed Range	p (t-test)	p (Wilcoxin)	p (ANCOVA)
Self-reported health	3.26 (0.890)	3.26-3.39	1-5	3.20 (1.05)	3.05-3.35	1-5	.548	.543	.403
Pain VNS	4.74 (3.26)	4.29-5.19	0-10	4.50 (3.14)	4.05-4.95	0-10	.465	.625	.402
Shortness of Breath VNS	3.35 (3.04)	2.93-3.77	0-10	3.91 (3.16)	3.46-4.36	0-10	.074	.081	.254
Fatigue VNS	6.11 (2.53)	5.76-6.46	0-10	5.94 (2.69)	5.56-6.33	0-10	.530	.489	.421
MD visits	4.69 (5.29)	3.96-5.42	0-35	5.59 (11.6)	3.94-7.24	0-150	.325	.269	.306
ER visits	0.534 (1.43)	0.338-0.731	0-10	0.938 (3.57)	0.429-1.45	0-32	.146	.330	.120
Hosp nights	1.82 (8.83)	0.600-3.04	0-10	2.04 (13.7)	0.090-3.99	0-10	.849	.812	.875
Hospitalizations	0.319 (1.07)	0.172-0.466	0-90	0.307 (0.989)	0.167-0.448	0-180	.913	.819	.883
Illness intrusiveness	13.1 (4.64)	12.4-13.7	4-21	13.1 (4.52)	12.5-13.8	3-21	.892	.893	.897
Health distress	2.25 (0.297)	2.09-2.44	0-5	2.46 (1.30)	2.28-2.65	0-5	.116	.111	.193
Self efficacy	5.92 (1.24)	5.60-6.23	1-10	6.01 (2.32)	5.68-6.34	1-10	.687	.684	.580
HAQ disability	0.284 (0.390)	0.230-0.338	0-1.63	0.254 (0.393)	0.198-0.310	0-2.13	.450	.306	.363
Doctor comm.	3.02 (1.27)	2.85-3.20	0-5	3.13 (1.19)	2.96-3.30	0-5	.379	.487	.208
Relaxation	1.70 (3.65)	1.19-2.20	0-20	1.61 (3.29)	1.14-2.08	0-25	.804	.461	.820
Range of motion exercise	37.1 (51.1)	30.1-44.2	0-180	34.4 (46.8)	27.7-41.0	0-180	.577	.793	.667
Aerobic exercise	84.5 (96.7)	71.1-97.8	0-420	86.6 (102.9)	71.9-101.2	0-585	.836	.580	.770

**Table 3:** Reliability

Variable	Internet Test-retest Reliability, N=30		I-C Reliability, Cronbach alpha	
	Pearson r	Spearman r	Web (N=204)	Questionnaire (N=191)
Self-reported health	.884	.890	single item	single item
Pain VNS	.847	.832	single item	single item
Shortness of Breath VNS	.968	.940	single item	single item
Fatigue VNS	.864	.827	single item	single item
MD visits	.784	.783	single item	single item
ER visits	.999	1.000	single item	single item
Hosp nights	.992	.999	single item	single item
Hospitalizations	1.000	1.000	single item	single item
Illness intrusiveness	.869	.880	.668	.658
Health distress	.935	.930	.935	.931
Self efficacy	.906	.870	.912	.922
HAQ disability	.930	.931	.874	.879
Doctor communication	.874	.865	.775	.750
Relaxation	.684	.802	single item	single item
Range of motion exercise	.829	.878	single item	single item
Aerobic exercise	.765	.921	n/a	n/a

## Discussion

The group randomized to mailed questionnaires required more follow-up effort than those randomized to Internet questionnaires. Although there was a slightly higher return rate among the Internet group (87.5% versus 83.1%), that difference was not statistically significant. We can conclude that among a population recruited through the Internet, participation among those assigned to the Internet was at least as good as, if not better than, participation among those assigned mailed questionnaires, with less recruitment effort required. However, the same results might not have occurred among a population less familiar and less comfortable with the Internet.

Our sample was a volunteer (convenience sample) drawn from a population who had access to and who were familiar with the Internet. Thus the results particularly apply to such populations and may not be representative of a broader-based population. However, Gosling et al [19] have argued that Internet samples may actually be more representative than traditional samples. Paper-and-pencil questionnaires will remain useful in target populations who have limited experience with or access to the Internet, while Internet surveys may allow researchers to reach more geographically diverse populations with less expense.

The results showed few differences between Internet-based and mailed paper questionnaires. None were significantly different at the .05 level when appropriate tests were used. With 16 instruments tested, we might expect to find several significantly different at the .20 level or lower, even if the two groups were more or less identical in how they answered the questions. And we did find a consistent difference at that level for one variable, health distress. Further testing on health distress might be warranted to determine if this slight trend toward Internet-based questionnaires showing more health distress could be replicated. Health distress did have high internal consistency reliability and high test-rest reliability, which was nearly identical for both Internet-based and mailed questionnaires. Thus we can be confident that health distress is reliable when administered via the Internet, even though there may be a possibility of slight differences in the normative values of the two different modes of administration.

Shortness of breath also showed a trend toward being significantly different when evaluated using bivariate statistics (t-tests and Wilcoxin). But when the presence of asthma was included as a covariate in an analyses of covariance model, the significance rose to a level indicating minor differences. This was because of the higher level of asthma in the mailed questionnaire group compared to the Internet group. The Shortness of Breath Visual Numeric Scale might also benefit from being tested in a new sample that did not show differences in asthma between the two randomized groups.

In summary, the instruments administered via the Internet appear to be reliable and appear to be answered similarly to the way they are answered when they are administered via mailed paper questionnaires.
